# Mortality in Acromegalic Patients: Etiology, Trends, and Risk Factors

**DOI:** 10.7759/cureus.14265

**Published:** 2021-04-02

**Authors:** Fatmah S Alhawyan

**Affiliations:** 1 Internal Medicine Endocrinology, Armed Forces Hospital, Abha, SAU

**Keywords:** acromegaly, mortality, growth hormone, risk factors

## Abstract

Although acromegaly has been associated with increased mortality rates, evidence shows that the application of the recent treatment modalities has reduced the risk of death in these patients, being comparable with the general population. As a result of the changing trends regarding mortality, the aim is to review the current literature to create enough evidence about the recent trends of mortality in patients with acromegaly. Moreover, this review aims to identify the possible etiology and the related risk factors. Old studies have reported that cardiovascular complications were the major etiology for death among acromegalic patients. However, recent studies showed that malignancies-induced complications might be the leading factor, while other studies reported that cardiovascular complications are still the main culprit. Nonetheless, the recently estimated risk is similar to that in the general population. Studies reported a decrease in mortality rates among patients with acromegaly within the last decades, which is probably attributable to the recent changes in the management updates, the less frequent exposure to radiotherapy and focus on the common cardiovascular disorders associated with the disease. This review also found that exposure to radiotherapy, old age, hypopituitarism, active acromegaly, and high growth hormone (GH) levels are significant predictors of mortality in acromegalic patients. In conclusion, more effort should be made to decrease GH to favorable levels based on the recent guidelines.

## Introduction and background

Acromegaly develops as a result of high levels of growth hormone (GH) and insulin-like growth factor-I (IGF-I) leading to many chronic systemic complications. The main etiology in 98% of these events is the GH-secreting type pituitary adenoma [1, 2]. The disease has been found to impact both genders and is commonly diagnosed within the 40-50 age range. However, evidence shows that the diagnosis of the disease might be delayed up to 5-10 years after the first signs and symptoms [3, 4]. It has been estimated that three or four cases of acromegaly per one million population develop yearly, and the incidence seems to be stable over the years [4]. On the other hand, a previous nationwide Icelandic study reported that the incidence has increased up to seven folds in the last decade as compared to the period between 1955 and 1964 [5]. Recent studies have also reported that the prevalence of acromegaly seems to be increasing [4-6]. This has been a point of debate among studies.

Many complications have been reported with acromegaly. These include metabolic, such as hyperlipidemia, hyperglycemia and insulin resistance, cardiovascular, respiratory, neoplastic osteoarthropathy and hypopituitarism complications. Other adverse events that can occur to acromegalic patients include vertebral fractures and the possible reduction in the quality of life [6-8]. Many etiologies have been reported to be involved in the mortality of these patients, with cardiovascular complications being the commonest [6]. However, more recent studies reported that other etiologies might be involved as the risk and severity of cardiovascular disorders in these patients has recently decreased [9-11]. Although the disease is still associated with increased mortality rates, evidence shows that the application of the recent treatment modalities has reduced the risk of death in these patients, being comparable with the general population [11, 12].

Management that is focused on mortality reduction is also an important indicator for the mortality trends and prevalence in patients with acromegaly. Conventional radiotherapeutic approaches have been marked as a third-line treatment modality secondary to medical and surgical approaches [13, 14]. Recent evidence within the last two decades shows that the mortality rates of acromegaly have been normalized secondary to the novel management modalities, where radiotherapy is used less frequently [9, 14]. As a result of the changing trends regarding mortality, in the present study, it aims to review the current literature aiming at creating enough evidence about the recent trends of mortality in patients with acromegaly. Moreover, the study aims to identify the possible etiology and the related predicting factors.

## Review

Mortality in patients with acromegaly

Evidence in the literature supports the strong association between acromegaly and the risk of mortality. A previous investigation by Colao et al. [6], that collected related reports that were previously inaugurated until 2004, reported that acromegaly increased the risk of mortality in the affected patients, and 60% of the cases were attributable to cardiovascular complications, while 25% and 15% were complicated by respiratory diseases and malignancies, respectively. In recent decades, many studies have been published, investigating the effect of the new management modalities on the reported mortality rates among these patients.

Etiology of mortality

Colao et al. reported that death in patients with acromegaly mainly occurs secondary to the presence of cardiovascular complications [6]. However, evidence from more recent studies shows that the intensity and severity of these complications have been significantly reduced over time which affected the mortality rate among these patients [9, 11, 12, 15-17]. For instance, previous studies have demonstrated that death in acromegalic patients was mainly attributable to the presence of underlying malignancies followed by cardiovascular complications, and the mortality rates did not significantly differ from the reported causes and rates compared to the general population [9, 11, 12, 17]. This is supported by a recent review by Gadelha et al. that showed that the presence of malignancies was the most common cause of death within the last decade in acromegaly [18]. Ritvonen et al. reported that in 34% of the included patients, cardiovascular complications were the cause of death followed by the presence of malignancies in 27% of the included patients [16]. Sherlock et al. also reported that the main causes of mortality among their patients were cardiovascular, respiratory, and cerebrovascular diseases, while malignancies were not reported among any of these patients [19]. The previous Finnish study in 2016 by Ritvonen et al. reported that the etiology of mortality among patients with acromegaly has significantly changed over the last few decades [16]. The authors reported that the development of cardiovascular complications was the major etiology of mortality within the earlier decades, while complications secondary to malignancies were the major attributable causes of death within the following two decades. Esposito et al. also reported that circulatory diseases followed by malignancies were the commonest causes of death in their population of acromegaly [20]. The authors also reported that mortality secondary to infectious diseases has increased while cardiovascular diseases have increased within the female patients.

Trends of mortality: what has changed in the recent decades

Before 1995, studies showed that the risk of acromegaly-induced mortality increased by two to three folds [21-23]. In 2008, meta-analyses studies showed that these rates were remarkably reduced. One meta-analysis by Holdaway et al. [24] that extracted data from 18 studies and followed patients with acromegaly from 1965 to 2008 reported that the standardized mortality ratio (SMR) was estimated to be 1.7 [95% Confidence interval (CI): 1.5-2.0]. Moreover, they reported that before 1984, the SMR was 2.2 (95% CI: 1.8-2.8), which was significantly reduced to 1.3 (95% CI: 1.1-1.6) according to studies after this period. The other meta-analysis that was conducted by Dekkers et al. included 16 studies between 1970 and 2005 and estimated the SMR to be 1.92 (95% CI: 1.62-1.83) [25]. Besides, the authors have analyzed their data based on two-time periods similar to the previous meta-analysis and found that the SMR was significantly reduced in patients with acromegaly from 2.11 to 1.62, before and after 1995, respectively. This is probably due to the applied management modalities for these patients before and after these periods as the authors reported that radiotherapy was the main used modality for these patients before 1995.

After these two meta-analyses, many other studies have also been published investigating similar outcomes. Many observational studies have reported that the SMR to acromegalic patients was found to be similar to that of the general population [6, 9, 11, 12, 17]. This was indicated by the Italian study in 2012 by Arosio et al. [12] that reported a SMR of 1512 patients that suffered from acromegaly at 1.13 (95% CI: 0.87-1.46). Moreover, within the investigations that showed high SMR rates, the rates were seemingly normal after adjustment of the approached management modality. Varadhan et al. reported a significantly reduced SMR from 1.6 to 1.0, when they divided patients into two groups, before and after 1994, which supports that the SMR is significantly affected by the approached management modality [26]. Esposito et al. also estimated a high SMR of 2.79 (95% CI: 2.43-3.15), which was significantly reduced to 0.98 and 0.45 when they analyzed patients that were treated with surgery and medical therapy combined or with surgery alone, respectively [20]. The same findings were also reported in a multinational study that compared two Italian and Bulgarian populations and found that the estimated SMR for the two cohorts were significantly different based on the approached management modalities for the included patients [15].

Associated factors

Many previous investigations have discussed the ability of certain factors to predict the death in patients with acromegaly. McCabe et al. indicated that having a history of radiotherapy exposure and old age were the most commonly reported predicting factors for mortality in these patients [27]. Bogazzi et al. also reported that radiotherapy was a significant factor in predicting mortality with an estimated hazard ratio of 34.25 [17]. The reported increased risk of mortality in these patients secondary to radiotherapy management is probably due to the development of cerebrovascular disorders [15]. It is worth mentioning that the effect of novel radiotherapeutic modalities, including the fractionated stereotactic radiotherapy and stereotactic radiosurgery on mortality rates in acromegalic patients is scarce and could not be evaluated in this study. In addition to radiotherapy and old age, previous studies have also demonstrated that the risk of mortality significantly increases if the disease was associated with hypopituitarism [19, 26, 27]. For instance, Sherlock et al. previously reported that the SMR for their acromegalic population significantly increased to 2.1 (95% CI: 1.6-2.7), and 2.5 (95% CI: 1.9-3.2) after they have noticed significant differences in the gonadotropin and ACTH hormonal levels, respectively [19]. Notably, the authors reported that only ACTH was associated with the increased risk of mortality in these patients after adjustment of several variables such as sex, age, and radiotherapy with an estimated risk ratio (RR) of 1.7 (95% CI: 1.2-2.5). The dosage system of hydrocortisone replacement therapy had also a significant role in predicting mortality in patients with acromegaly. Sherlock et al. reported that a daily dose of 25-30 mg was associated with a RR of 1.6 (95% CI: 1.1-2.4), which was significantly increased up to 2.9 (95% CI: 1.4-5.9) in patients that received doses over 30 mg per day [19]. Previous studies have also demonstrated the presence of active acromegaly is a significant indicator for death [12, 24-27]. Although Arosio et al. recently reported that the SMR for patients with acromegaly has recently normalized after the new treatment modalities, they reported that the analysis of patients that suffered from active acromegaly only showed that the SMR was significantly increased to 1.93 (95% CI: 1.34-2.70) [12]. Therefore, creative approaches should be sought to appropriately apply disease control strategies to reduce the mortality in acromegalic patients.

Acromegaly disease control

The appropriate control of acromegaly disease has also changed over decades. McCabe et al. indicated that maintaining favorable GH levels is significantly associated with a reduced risk of mortality in patients with acromegaly [27]. The previous meta-analysis by Holdaway et al. reported that the estimated SMR for acromegalic patients was associated with the estimated levels of GH in the collected studies [24]. They reported that the SMR was significantly reduced from 1.9 (95% CI: 1.5-2.4) to 1.1 (95% CI: 0.9-1.4) following a reduction in GH levels from >2.5 µg/L to <2.5 µg/L, respectively. Notably, the authors reported that the included studies have used the radioimmunoassay for measurement of the GH levels in their patients, and using modern assays to measure the GH levels indicated that the lower levels are associated with reduced risk of mortality. This is consistent with the recent investigations which reported that improved clinical outcomes were significantly associated with reduced GH levels to <1.0 µg/L [12, 15, 17, 24, 26-29]. Therefore, it has recently been recommended by the Endocrine Society that clinicians should target a GH level less than 1.0 µg/L to achieve better prognostic outcomes in patients with acromegaly [13]. On the other hand, McCabe et al. reported that such guidelines might not necessarily reflect the cumulative assessment of GH levels and their effects on the clinical outcomes [27]. The cumulative effect was investigated by Sherlock et al. [30] in a time-dependent approach and was found to be a better approach for the assessment of the prognostic outcomes as the authors reported that GH levels <1.0 µg/L were associated with a RR of 1.8 for mortality, while a GH levels <0.5 µg/L were associated with a lower RR of 1.5 among the investigated patients with acromegaly by using the time-dependent approach for assessment of GH levels. Therefore, this approach should be recommended to obtain better prognostic outcomes and reduce the risk of mortality among these patients [30]. Moreover, IGF-I levels in predicting the clinical outcomes in acromegalic patients has been poorly reported in the literature [27]. The association between IGF-I and mortality in patients with acromegaly has been reported to be significant in some studies [9, 12, 17] and insignificant in others [15, 16, 19, 26, 28, 30]. Holdaway et al. estimated that the SMR was significantly higher in patients with elevated IGF-I (SMR: 2.5; 95% CI: 1.6-4.0) than patients with normalized levels of IGF-I (SMR: 1.1; 95% CI: 0.9-1.4) [24]. However, they also reported that only the GH level was found to be the only significant factor to predict mortality in patients with acromegaly, according to the results of the multivariate analysis.

## Conclusions

The present study focused on the recent literature that investigated mortality among patients with acromegaly and the associated risk factors. We have noticed that studies have agreed that mortality rates have been reduced among patients with acromegaly within the last decades, which is probably attributable to the recent changes in the management modalities as the less frequent exposure to radiotherapy and the more frequent management of cardiovascular disorders, which has been marked as the most common cause of mortality in these patients.
